# The incidence, risk factors, and clinical outcomes of rhabdomyolysis associated with fenoverine prescription: a retrospective study in South Korea (1999–2014)

**DOI:** 10.1186/s40360-020-00408-3

**Published:** 2020-04-25

**Authors:** Junhyeong Cho, Jeonggu Na, Eunjin Bae, Tae Won Lee, Ha Nee Jang, Hyun Seop Cho, Se-Ho Chang, Dong Jun Park

**Affiliations:** 1 Department of Internal Medicine, Changwon Gyeongsang National University Hospital, Changwon, South Korea; 2 Department of Internal Medicine, College of Medicine, Gyeongsang National University, Jinju, South Korea; 3 Department of Internal Medicine, Gyeongsang National University Hospital, Jinju, South Korea; 4 Institute of Health Science, Gyeongsang National University, Jinju, South Korea

**Keywords:** Rhabdomyolysis, Risk factors, Drugs, Epidemiology, Fenoverine

## Abstract

**Background:**

Fenoverine is a spasmolytic drug that has been used to treat abdominal pain. Although sporadic case reports or case series of rhabdomyolysis associated with fenoverine have been published, there are no studies evaluating the incidence, risk factors, and clinical outcomes of rhabdomyolysis associated with fenoverine prescription.

**Methods:**

We retrospectively reviewed the medical records of 22 patients admitted with rhabdomyolysis associated with fenoverine from January 1999 to December 2014, while excluding other well-known risk factors of rhabdomyolysis. This period was subdivided into two periods, January 1999–December 2007 and January 2008–December 2014. We analyzed the clinical and laboratory characteristics, and the prognosis of fenoverine associated with rhabdomyolysis for these times.

**Results:**

The incidence of rhabdomyolysis associated with fenoverine was 0.27% during the total period (22/8257), 0.34% in the first period (18/5298), and 0.14% in the second period (4/2959) (*p* < 0.001). Rhabdomyolysis occurred in 19 liver cirrhosis (LC) patients (2.03%), whereas only 3 cases (0.04%) occurred in non-LC patients (*p* < 0.001). Drug duration, total dose, muscle enzymes, and clinical characteristics were not different between the LC and non-LC groups. Acute renal failure (ARF) occurred in 5 patients in the LC group and 2 patients in the non-LC group (*p* = 0.227). Severity of hepatic derangement according to the Child-Pugh classification was not different between the ARF group and non-ARF group (*p* = 0.227). Four patients died, having complications of oliguric ARF (*p* = 0.005) and underlying severe LC (*p* = 0.017). Higher serum lactate dehydrogenase, blood urea nitrogen, creatinine, and potassium levels but lower serum sodium levels were found in the group that died (*p* = 0.001).

**Conclusions:**

Physicians should carefully prescribe fenoverine because it may cause rhabdomyolysis, especially in patients with LC.

## Background

Rhabdomyolysis is the release of muscular cell constituents into the extracellular fluid and its circulation after a striated muscle injury [1–3]. The degree of rhabdomyolysis ranges from a subclinical rise in creatinine kinase (CK) to acute kidney injury [4–7]. The medical causes of rhabdomyolysis include drugs or toxins, muscle hypoxia, metabolic and endocrine disorders, infections, dehydration, temperature alterations, drugs, and electrolyte disturbances [4–9]. Regular and illegal drugs can cause rhabdomyolysis [4, 9]; the most frequent cause of drug-induced rhabdomyolysis is the administration of HMG-CoA reductase inhibitors [9–12].

Fenoverine is a drug with a phenothiazine structure that inhibits calcium channel currents. It has a non-atropine-like spasmolytic action on muscles and is prescribed for irritable bowel syndrome with pain and abnormal intestinal transit. It has been used widely in continental Europe and Latin America to treat gastrointestinal and gynecological spasmodic disorders since 1979 [13, 14]. In Korea, it has been used since studies showed its clinical efficacy and safety for irritable bowel syndrome in 1989. However, in several cases it has been reported as a cause of rhabdomyolysis [15–18]. One report showed that 45 cases of rhabdomyolysis were attributed to this medication over the course of 5 years [19]. The incidence of reported cases has been estimated to be around 1 per 1 million days of treatment [19]. Well-known risk factors for fenoverine-induced rhabdomyolysis are hepatic dysfunction, renal dysfunction, and concomitant use of lipid-lowering drugs [18–22]. The authors intermittently have experienced rhabdomyolysis which might be associated with fenoverine medication and retrospectively undertook this study to evaluate the clinical and laboratory characteristics and prognosis of fenoverine-associated rhabdomyolysis.

Initially, fenoverine associated rhabdomyolysis, which occurred first period (January 1999 –December 2007) was published in the 2008 master’s thesis at Gyeongsang National University as case series. We again complied and analyzed the data and wrote a manuscript and presented “Clinical characteristics of rhabdomyolysis associated with fenoverine treatment” as a poster presentation (No-MP340) at the ERA-EDTA 50th Congress held in Istanbul in 2013. In that presentation, we retrospectively investigated patients with rhabdomyolysis associated with fenoverine at our hospital from January 1999 to December 2007. Based on our work, it was naturally recognized during the process of research in 2008 that fenoverine might be related with rhabdomyolysis and it was recommended that fenoverine prescriptions be limited in patients from 2008 onwards in our institution, especially patients with poor liver function. Therefore, we investigated changes in fenoverine prescriptions and compared epidemiologic changes in rhabdomyolysis associated with fenoverine before (January 1999–December 2007) and after 2008 (January 2008–December 2014).

## Methods

We initially searched for 8257 patients who were prescribed fenoverine through prescription code and selected patients with the disease code of “Rhabdomyolysis” among them from January 1999 to December 2014 at our institution. Patients without temporal relationship and considered to have other causative factors except for fenoverine were excluded. We enrolled total 22 admitted patients who meet the above criteria. We divided this time into two periods, the first from January 1999 to December 2007 and the second from January 2008 to December 2014. All patients had documented medical histories and underwent a medical interview as well as a routine general physical examination. Clinical and laboratory data were reviewed for characteristics of fenoverine associated with rhabdomyolysis.

Rhabdomyolysis was defined as a serum CK concentration above 1000 IU/L (normal level, 0–170 IU/L) accompanied by clinical symptoms such as muscle pain, weakness, dark urine, and oliguria at admission [23]. We excluded patients with other causes of rhabdomyolysis, including infection, trauma, severe physical exercise, burns, toxins, drugs other than fenoverine, electrolyte abnormalities, alcohol consumption, metabolic derangements such as diabetic ketoacidosis, and endocrine abnormalities as far as possible based on medical records. Patients with evidence of an acute myocardial infarction or acute cerebral vascular accident were also excluded from the analysis.

A diagnosis of liver cirrhosis (LC) was based on clinical, laboratory, and radiological data in patients who had predisposing factors such as a history of persistent alcohol intake, chronic viral infection (hepatitis B or C), or cryptogenic symptoms. LC patients were classified into Child-Pugh class A–C, employing five known clinical measures of liver disease (total bilirubin, serum albumin, prothrombin time, international normalized ratio, ascites, and hepatic encephalopathy). Child-Pugh “C” was arbitrarily defined as “Severe hepatic derangement,” which was one of the statistical parameters used to evaluate the association of hepatic derangement and acute renal failure (ARF) occurrence and survival. We also divided the study group patients into subgroups according to whether ARF occurred (ARF group) or not (non-ARF group). ARF was defined as an increase in serum creatinine of more than 50% over the baseline value. The death rate was also compared between the two groups.

The study protocol was approved by the Institutional Review Board of Gyeongsang National University Hospital (IRB no. 2013–03-018).

### Statistical analysis

All measurement values are expressed as the mean ± standard deviation (SD). We used Pearson’s chi-square and Fisher’s exact test to analyze qualitative differences. The nonparametric Mann-Whitney U test was performed for the comparison of means in small samples of similar variance. A *p*-value less than 0.05 was taken to indicate statistical significance. Statistical analysis was carried out using SPSS for Windows software (V.21.0; IBM Corp., Armonk, NY, USA).

## Results

### Epidemiologic changes in fenoverine-associated rhabdomyolysis between the two periods

**Fig. 1:**
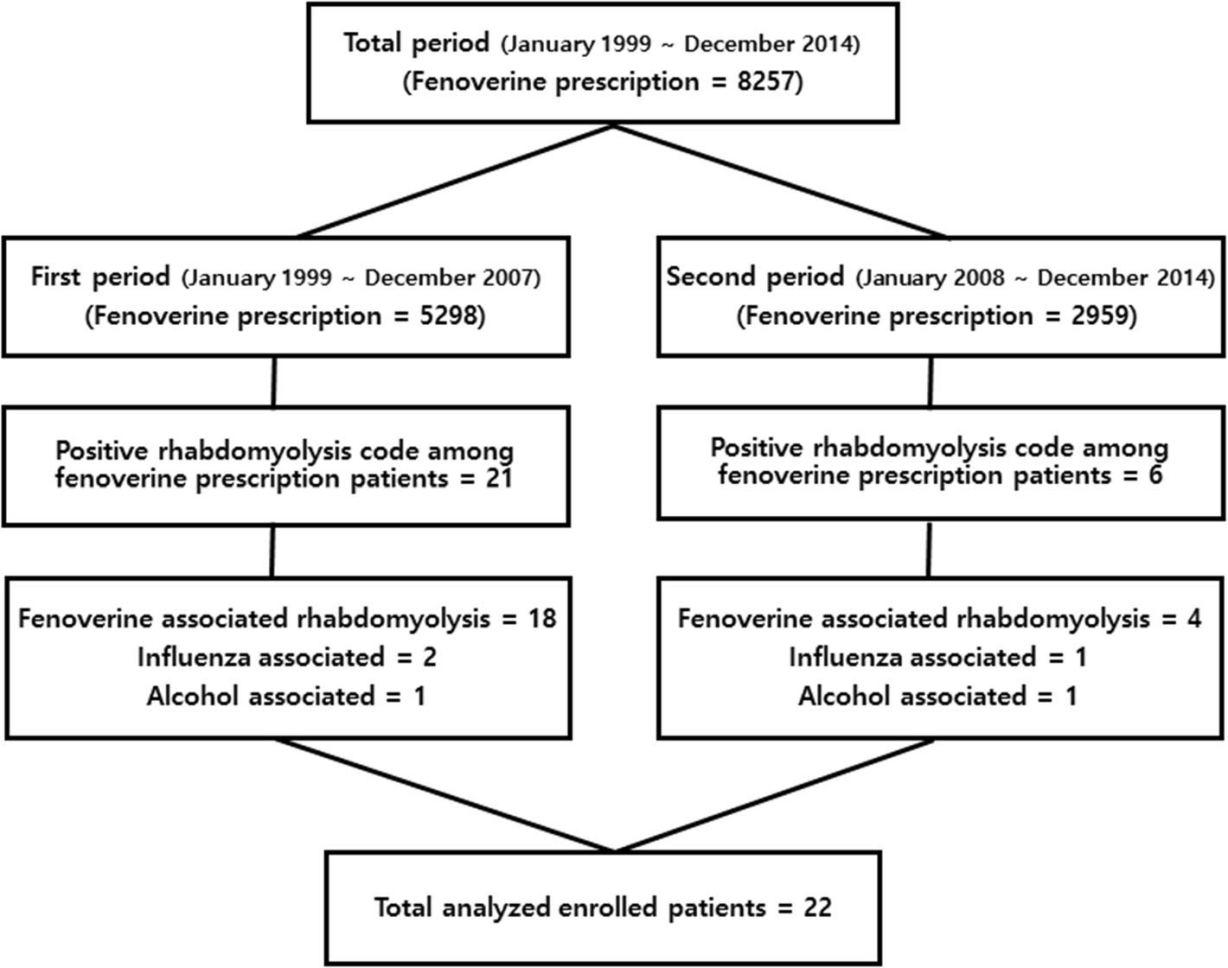
Overall workflow of patient’s enrollment

**Fig. 2:**
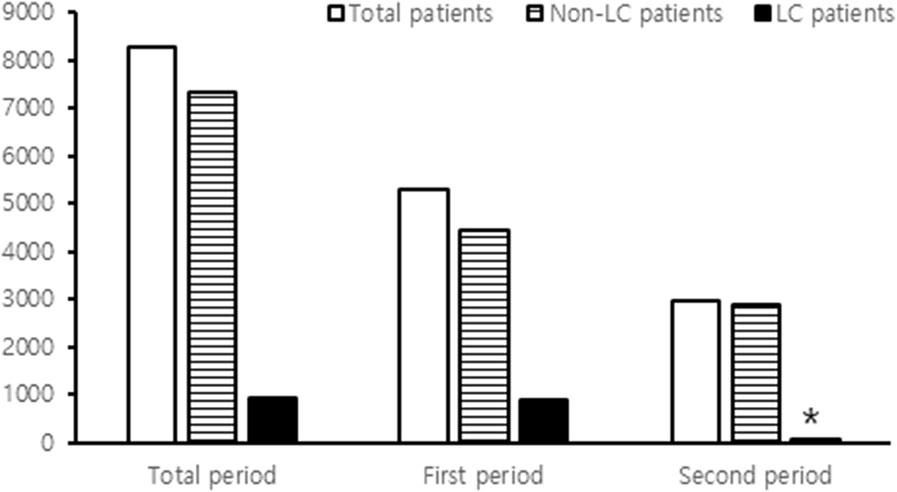
The change of prescription number according to each period (*p < 0.001, compared with first period)

**Fig. 3:**
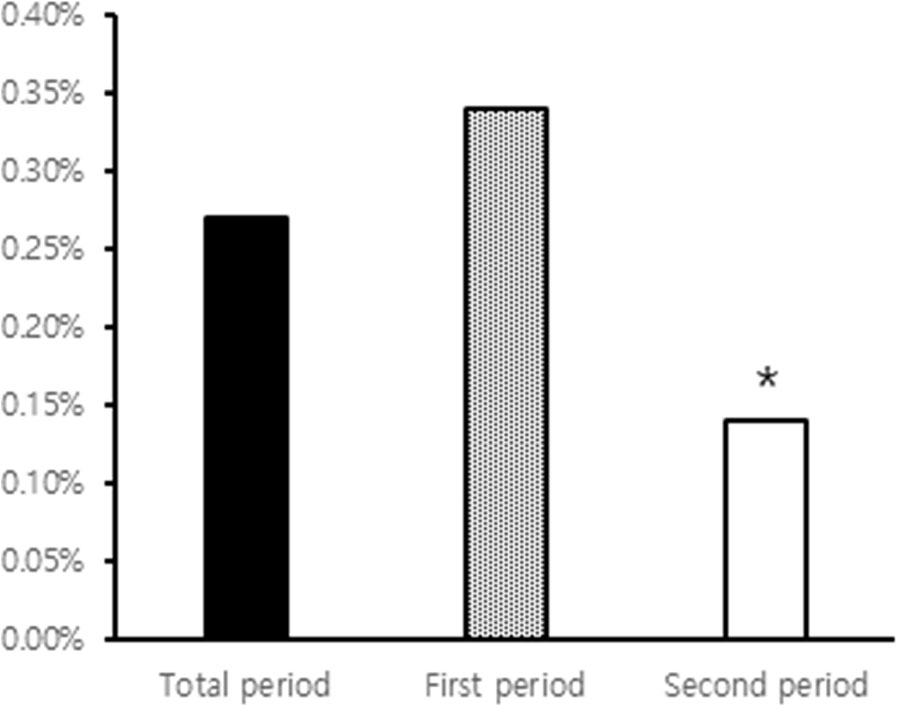
The change in the incidence of fenoverine associated rhabdomyolysis according to each period (*p < 0.001, compared with first period)

Twenty-seven cases of rhabdomyolysis have occurred in 8257 patients prescribed fenoverine, 21 cases in the first period and 6 cases in the second period. Five cases were excluded in analysis due to other etiologies (Fig. 1). Fenoverine was prescribed to 589 patients per year in the first period and 423 patients per year in the second period. Fenoverine was prescribed to 938 patients with liver disease (11.4%); 16.5% in the first period (874/5298) and 2.2% in second period (64/2959) (*p* < 0.001) (Fig. 2). The incidence of rhabdomyolysis associated with fenoverine was 0.27% during the total period (22/8257), 0.34% in the first period (18/5298), and 0.14% in the second period (4/2959) (*p* < 0.001) (Fig. 3). 19/22 patients with fenoverine who developed rhabdomyolysis had liver disease (2.03%), 16 in the first period and 3 in the second period, with only 3 occurrences (0.04%) in patients with liver disease (*p* < 0.001).

### Clinical and laboratory characteristics of fenoverine associated with rhabdomyolysis

Twenty-two patients were admitted to our hospital with rhabdomyolysis associated with fenoverine treatment. Fenoverine was prescribed to these patients because of abdominal cramping. Overall, 14 patients were male; the mean age was 58 years. The average duration and total dose during treatment was 13.9 days and 4072 mg, respectively. Only one patient had diabetes. No patient had underlying hypertension. In total, 20 patients had muscle pain and 9 complained of muscle weakness. Four patients had oliguria and three had dark urine. The etiology of LC was as follows: hepatitis B (*n* = 12), hepatitis C (*n* = 2), alcohol (*n* = 4), and cryptogenic (*n* = 1). The patient number in Child-Pugh classes ‘A’, ‘B’, and ‘C’ was three, seven, and nine, respectively (Table 1).

**Table 1:** Baseline characteristics of total patients

Clinical and laboratory parameters	Total patients (*n* = 22)
Age	58.1 ± 9.7
Sex	
Male (%)	14 (63.6)
Female (%)	8 (36.4)
Drug duration (days)	13.9 ± 8.8
Drug total dose (mg)	4072.7 ± 2597.7
WBC (× 10^3^/*u*L)	7.0 ± 3.7
CK (U/L)	21,104.0 ± 5.0
LDH (U/L)	2430.5 ± 1623.1
AST (U/L)	1118.3 ± 635.3
ALT (U/L)	383.3 ± 234.5
Albumin (mg/dL)	2.8 ± 0.6
BUN (mg/dL)	29.8 ± 26.1
Creatinine (mg/dL)	1.9 ± 2.1
Sodium (mmol/L)	135.6 ± 6.5
Potassium (mmol/L)	4.6 ± 0.7
Hospital days	21.2 ± 9.7
Clinical signs and symptoms	
Muscle pain (%)	20 (90.9)
Muscle weakness (%)	9 (40.9)
Oliguria (%)	4 (18.2)
Dark urine (%)	3 (13.6)
DM (%)	1 (4.5)
ARF (%)	7 (31.8)
Death (%)	4 (18.2)
Child-Pugh classification	
A (%)	3 (15.8)
B (%)	7 (36.8)
C (%)	9 (47.4)
Etiology of LC	
Hepatitis B (%)	12 (63.2)
Hepatitis C (%)	2 (10.5)
Alcohol (%)	4 (21.1)
Cryptogenic (%)	1 (5.2)

*WBC* White Blood Cell, *CK* Creatine Kinase, *LDH* Lactate Dehydrogenase, *AST* Aspartate Transaminase, *ALT* Alaninine Transminase, *BUN* Blood Urea Nitrogen, *DM* Diabetes Mellitus, *ARF* Acute Renal Failure, *LC* Liver Cirrhosis

### Comparison of clinical and laboratory characteristics between the LC and non-LC groups

Age was not statistically different between the groups. The total drug dose was 3831 mg in the LC group and 5600 mg in the non-LC group (*p* = 0.284). The time to admission after medication was 13.2 and 18.7 days, respectively (*p* = 0.328). There were no clinical symptom differences between the two groups (e.g., muscle pain, muscle weakness, oliguria, or dark urine). The mean duration of hospitalization was longer for LC patients, although there was no statistical significance (24 vs. 11 days, *p* = 0.081). ARF occurred in 5 patients (26.3%) in the LC group and 2 patients (66.7%) in the non-LC group (*p* = 0.227). The serum sodium level was lower in the LC group compared to the non-LC group, though the difference was not statistically significant (*p* = 0.186). The serum blood urea nitrogen (BUN) levels and creatinine levels were not different between the two groups (*p* = 0.141 and 0.075, respectively). There were no significant differences in muscle enzyme serum levels such as CK, lactate dehydrogenase (LDH), aspartate transaminase (AST), and alanine aminotransaminase (ALT) (*p* = 0.984, 0.466, 0.518, and 0.606, respectively) (Table 2).

**Table 2:** Clinical and laboratory parameters in LC group and non-LC group

Clinical and laboratory parameters	LC group (*n* = 19)	Non-LC group (*n* = 3)	*P*
Age	57.4 ± 9.9	63.0 ± 7.6	0.361
Sex			0.273
Male (%)	11 (57.9)	3 (100)	
Female (%)	8 (42.1)	0 (0)	
Drug duration (days)	13.2 ± 8.6	18.7 ± 10.5	0.328
Drug total dose (mg)	3831.6 ± 2513.8	5600.0 ± 3152.2	0.284
WBC (× 10^3^/uL)	6.2 ± 3.4	12.0 ± 1.6	0.009
CK (U/L)	21,132.1 ± 16,865.7	20,930.0 ± 9363.7	0.984
LDH (U/L)	2416.1 ± 1578.6	1716.0 ± 753.2	0.466
AST (U/L)	1088.3 ± 638.2	1403.5 ± 748.8	0.518
ALT (U/L)	374.5 ± 244.2	467.5 ± 105.4	0.606
Albumin (g/dL)	2.7 ± 0.5	3.5 ± 0.8	0.034
BUN (mg/dL)	26.5 ± 22.9	50.6 ± 40.9	0.141
Creatinine (mg/dL)	1.6 ± 1.8	3.9 ± 3.5	0.075
Sodium (mmol/L)	134.9 ± 6.5	140.3 ± 4.7	0.186
Potassium (mmol/L)	4.5 ± 0.7	4.9 ± 0.7	0.336
Hospital days	24.0 ± 9.5	11.0 ± 5.6	0.081
Clinical signs and symptoms			
Muscle pain (%)	17 (89.5)	3 (100)	1.000
Muscle weakness (%)	8 (42.1)	1 (33.3)	1.000
Oliguria (%)	2 (10.5)	2 (66.7)	0.371
Dark urine (%)	2 (10.5)	1 (33.3)	0.073
DM (%)	1 (5.2)	0	1.000
ARF (%)	5 (26.3)	2 (66.7)	0.227
Death (%)	4 (21.1)	0 (0)	0.603
Child-Pugh classification			
A (%)	3 (15.8)	NA	
B (%)	7 (36.8)	NA	
C (%)	9 (47.4)	NA	
Etiology of LC		NA	
Hepatitis B (%)	12 (63.2)	NA	
Hepatitis C (%)	2 (10.5)	NA	
Alcohol (%)	4 (21.1)	NA	
Cryptogenic (%)	1 (5.2)	NA	

*WBC* White Blood Cell, *CK* Creatine Kinase, *LDH* Lactate Dehydrogenase, *AST* Aspartate Transaminase, *ALT* Alaninine Transminase, *BUN* Blood Urea Nitrogen, *DM* Diabetes Mellitus, *ARF* Acute Renal Failure, *LC* Liver Cirrhosis

### Comparison of clinical and laboratory characteristics between the ARF and non-ARF groups

ARF developed in 31.8% of patients (7/22). Five patients had LC whereas two did not have LC. Age was not significantly different between the two groups. Initial ARF was present in five patients—three patients in the LC group, and two patients in the non-LC group. ARF newly occurred during hospitalization in two LC patients. The initial sodium level was significantly lower in the ARF group, whereas the potassium level was higher in the ARF group, compared with the non-ARF group (*p* = 0.020 and 0.001, respectively). Among the 19 LC patients, ARF occurred in one Child-Pugh class A and four Child-Pugh class C patients. The mean duration of medication was 15.9 and 13.0 days in the two groups (*p* = 0.494), and the mean dose of total drug dose was 4757 and 3753 mg, respectively (*p* = 0.412). There were no significant differences in muscle enzyme serum levels such as CK, LDH, AST, and ALT (*p* = 0.290, 0.282, 0.085, and 0.327, respectively) (Table 3). There was no significant difference between ARF and the severity of LC classified by Child-Pugh scoring in patients with rhabdomyolysis (*p* = 0.312).

**Table 3:** Clinical and laboratory parameters in ARF group and non-ARF group

Clinical and laboratory parameters	ARF group (*n* = 7)	Non-ARF group (*n* = 15)	*P*
Age	58.3 ± 10.5	58.1 ± 9.6	0.962
Sex			0.671
Male (%)	5 (71.4)	9 (60)	
Female (%)	2 (28.6)	6 (40)	
Drug duration (days)	15.9 ± 7.8	13.0 ± 9.4	0.494
Drug total dose (mg)	4757.1 ± 2345.8	3753.3 ± 2723.9	0.412
WBC (× 10^3^/uL)	9.4 ± 5.4	5.9 ± 2.0	0.034
CK (U/L)	26,467.8 ± 22,434.3	18,601.6 ± 11,893.8	0.290
LDH (U/L)	2836.0 ± 2497.6	2080.1 ± 712.3	0.282
AST (U/L)	1495.8 ± 718.3	967.3 ± 553.9	0.085
ALT (U/L)	464.9 ± 215.7	350.8 ± 240.8	0.327
Albumin (g/dL)	3.2 ± 0.6	2.7 ± 0.6	0.105
BUN (mg/dL)	56.7 ± 32.3	17.3 ± 6.9	< 0.001
Creatinine (mg/dL)	4.2 ± 2.6	0.8 ± 0.3	< 0.001
Sodium (mmol/L)	131.1 ± 6.5	137.8 ± 5.5	0.020
Potassium (mmol/L)	5.2 ± 0.8	4.3 ± 0.4	0.001
Hospital days	17.6 ± 9.8	22.9 ± 9.4	0.234
Clinical signs and symptoms			
Muscle pain (%)	7 (100)	13 (86.7)	0.545
Muscle weakness (%)	3 (42.9)	6 (40.0)	1.000
Oliguria (%)	4 (57.1)	0 (0)	0.005
Dark urine (%)	2 (28.6)	1 (6.7)	0.227
LC (%)	5 (26.3)	14 (93.3)	0.227
Child-Pugh classification			0.312
A (%)	0 (0)	3 (21.4)	
B (%)	1 (20.0)	6 (42.9)	
C (%)	4 (80.0)	5 (35.7)	
Severe hepatic derangement	4 (80)	5 (35.7)	0.376
Etiology of LC			0.830
Hepatitis B (%)	3 (60)	9 (64.3)	
Hepatitis C (%)	1 (20)	1 (5.3)	
Alcohol (%)	1 (20)	3 (25.1)	
Cryptogenic (%)	0 (0)	1 (5.3)	
Death (%)	4 (57.1)	0 (0)	0.005

*WBC* White Blood Cell, *CK* Creatine Kinase, *LDH* Lactate Dehydrogenase, *AST* Aspartate Transaminase, *ALT* Alaninine Transminase, *BUN* Blood Urea Nitrogen, *DM* Diabetes Mellitus, *ARF* Acute Renal Failure, *LC* Liver Cirrhosis

### Clinical outcomes

Four instances of patient mortality occurred (18.2%). All four deceased patients had complications of LC, and there were no deaths in the non-LC group (*p* = 0.603) (Table 1). Higher mortality occurred in the ARF group (*p* = 0.005) (Table 2). All deceased patients experienced hepatic and renal failure. All surviving patients recovered from ARF. There were no differences in clinical symptoms associated with rhabdomyolysis such as muscular symptoms, oliguria, and dark urine between the deceased and living groups (Table 4). Age, drug duration, mean drug dose, and hospital duration did not differ between the two groups. Factors that affected survival included LDH (*p* = 0.042), BUN (*p* = 0.001), creatinine (*p* = 0.003), sodium level (*p* = 0.001), potassium level (*p* = 0.019), and ARF occurrence (*p* = 0.005). The severity of liver disease classified by Child-Pugh scoring was not different between the two groups (Table 4).

**Table 4:** Clinical and laboratory characteristics in death group and survival group

Clinical and laboratory parameters	Death group (*n* = 4)	Survival group (*n* = 18)	*P*
Age	56.8 ± 12.5	58.4 ± 9.3	0.760
Sex			0.671
Male (%)	5 (71.4)	9 (60)	
Female (%)	2 (28.6)	6 (40)	
Drug duration (days)	15.0 ± 6.5	13.7 ± 9.4	0.792
Drug total dose (mg)	4500 ± 1944	3977.8 ± 2759.7	0.726
WBC (× 10^3^/uL)	9.0 ± 7.1	6.6 ± 2.7	0.254
CK (U/L)	27,111.3 ± 29,658.8	19,769.7 ± 12,078.8	0.416
LDH (U/L)	3681.5 ± 3142.2	2018.2 ± 710.6	0.042
AST (U/L)	1495.8 ± 718.3	967.3 ± 553.9	0.622
ALT (U/L)	420.3 ± 263.7	374.6 ± 235.1	0.736
Albumin (g/dL)	2.8 ± 0.3	2.8 ± 0.7	0.957
BUN (mg/dL)	64.7 ± 22.5	22.0 ± 20.1	0.001
Creatinine (mg/dL)	4.5 ± 1.9	1.3 ± 1.7	0.003
Sodium (mmol/L)	126.9 ± 3.8	137.6 ± 5.2	0.001
Potassium (mmol/L)	5.3 ± 0.9	4.4 ± 0.5	0.019
Hospital days	21.8 ± 11.6	21.1 ± 9.6	0.908
Clinical signs and symptoms			
Muscle pain (%)	7 (100)	13 (86.7)	1.000
Muscle weakness (%)	3 (42.9)	6 (40.0)	0.616
Oliguria (%)	4 (57.1)	0 (0)	0.135
Dark urine (%)	2 (28.6)	1 (6.7)	1.000
LC (%)	4 (100)	15 (83.3)	1.000
Child-Pugh classification			0.312
A (%)	0 (0)	3 (20.0)	
B (%)	0 (0)	7 (46.7)	
C (%)	4 (100)	5 (33.3)	
Severe hepatic derangement	4 (100)	5 (33.3)	0.017
Etiology of LC			1.000
Hepatitis B (%)	3 (75)	9 (60)	
Hepatitis C (%)	0 (0)	2 (13.3)	
Alcohol (%)	1 (25)	3 (20)	
Cryptogenic (%)	0 (0)	1 (6,7)	
ARF (%)	4 (100)	3 (16.7)	0.005

*WBC* White Blood Cell, *CK* Creatine Kinase, *LDH* Lactate Dehydrogenase, *AST* Aspartate Transaminase, *ALT* Alaninine Transminase, *BUN* Blood Urea Nitrogen, *DM* Diabetes Mellitus, *ARF* Acute Renal Failure, *LC* Liver Cirrhosis

## Discussion

Our study suggests that fenoverine treatment has correlation with rhabdomyolysis in patients with LC. The incidence of rhabdomyolysis was 2.03% in patients with LC and 0.04% in patients with normal liver function. This was accompanied by ARF in some cases (31.8%), and mortality was significantly more common in this case. Deceased patients had both ARF and LC.

Hepatic derangement is one of the most important underlying diseases in fenoverine-associated rhabdomyolysis [17–20]. However, there has been no report on the incidence in patients with fenoverine use. Our study demonstrates that the incidence of fenoverine-associated rhabdomyolysis was 2.03% in patients with LC under normal posology of fenoverine, and that the incidence tended to increase with the degree of liver failure according to Child-Pugh classification, though there was no significant difference. This might result from the small sample size of LC patients. Our study also shows that the less frequent prescription of fenoverine in the high-risk group resulted in a lower occurrence of rhabdomyolysis.

Chichmanian et al. reported 45 cases of rhabdomyolysis induced by fenoverine [19]. The mean time to admission in that study was 11 days while it was 14 days in our study. They proposed that rhabdomyolysis may be induced by a modification in the hepatic first-pass effect of fenoverine; in 30 cases, there was chronic liver disease or an association with hepatic inhibitory drugs and with highly metabolized drugs. Our case study reveals that 18 patients had various degrees of underlying LC, while 4 patients had normal liver function. Four patients also had no underlying disease and used no medications known to induce hepatic inhibition or drug metabolism [19]. However, they did not describe any other clinical features or laboratory characteristics as we did.

Rhabdomyolysis is a potentially deadly syndrome that is often unrecognized. There are many etiologies of rhabdomyolysis. Drug-induced rhabdomyolysis is the leading cause of this syndrome. One study reported that drug-induced rhabdomyolysis, including alcohol, comprised up to 81% of rhabdomyolysis cases [24]. This category of rhabdomyolysis is complicated by the fact that there are primary and secondary mechanisms of muscle cell injury [11]. Primary toxic-induced rhabdomyolysis is induced by a direct attack on skeletal myocyte function and integrity. Secondary effects result from predisposing risk factors such as local muscle compression in coma, prolonged seizures, trauma, and metabolic abnormalities [11, 24]. Chariot et al. also described two patients with fenoverine-associated rhabdomyolysis [18]. They suggested that focal muscle necrosis and regeneration on muscle biopsy might be associated with the muscular toxicity of fenoverine, and that ischemia may be one mechanism of fenoverine-induced rhabdomyolysis.

Jouglad et al. studied the predisposition for acute rhabdomyolysis attributed to fenoverine in 7 patients without underlying hepatic dysfunction [25]. They investigated all patients with the following tests: 31-phosphorus nuclear magnetic resonance spectroscopy, histopathological muscle findings, muscle contraction tests, and biochemical analyses of the muscle. All patients had muscle abnormalities and oxidative dysfunction induced by fenoverine. They concluded that there is a cause-and-effect link between underlying abnormalities and muscular cytolysis attributed to fenoverine. However, no patient had previously presented with clinical signs of a muscular disorder before fenoverine intake. This means that it is impossible to predict a patient’s predisposition to fenoverine toxicity before taking the medication if the patient has no underlying hepatic dysfunction.

The mortality rate in patients with rhabdomyolysis varies from 10 to 25%. The major cause of death is underlying disease progression [26, 27]. Kim et al. showed that the development of ARF and the presence of oliguria may be the most important prognostic factors [26]. Our results show that the mortality rate in fenoverine-induced rhabdomyolysis was 18.2%, and it was higher in the ARF group than in the non-ARF group. All deceased patients had poor hepatic function (Child-Pugh classification C) accompanied by oliguric ARF; no deaths occurred among patients with normal hepatic function. However, this does not mean that fenoverine was the direct cause of hepatic disease progression. Rather, disease progression and fenoverine administration likely occurred together by chance. Previous reports showed that fenoverine-associated rhabdomyolysis in patients with hepatic derangement led to death [18], while other studies of fenoverine-associated rhabdomyolysis did not shown any mortalities, even in patients with poor hepatic function [17, 19, 20, 22].

The most worrying limitation of our study is that the incidence of fenoverine-associated rhabdomyolysis might be underestimated because only hospitalized patients were included and subclinical presentations without common features such as myalgia, myoglobinuria, and those whose CK has risen slightly above the diagnostic criteria CK may have been overlooked. Another limitation is that we might not have been able to completely exclude other causes of rhabdomyolysis due to the characteristics of the retrospective analysis (depending on medical records). Other limitations also exist that enrolled patients were only limited to cases of rhabdomyolysis in patients who have been prescribed with fenoverine and diagnosed in our hospital. It was excluded from cases where patients were prescribed at our hospital and diagnosed with rhabdomyolysis at another hospital. Since we have searched and enrolled patients with prescription and disease codes, there is a possibility that data will be less accurate leading to underestimate the incidence of rhabdomyolysis. Another limitation of our study, which has relatively long retrospective study, includes that rhabdomyolysis patients in the second period could receive better cares resulting in the improvement of prognosis. Furthermore, the incidence in LC patients may be more accurate and higher than in patients with normal hepatic function because of frequent and periodic follow up at the hospital. Above all, The number of sampling size is so small that it is considered difficult to generalize the results of this research. However, it is meaningful that we included many patients taking fenoverine at a single center, especially LC patients.

## Conclusions

Fenoverine might be associated with rhabdomyolysis in patients with severe hepatic dysfunction and fatal in these patients with the occurrence of ARF. Therefore, physicians should carefully prescribe fenoverine in patients with LC.

## Data Availability

The datasets used and/or analyzed during this study are available from the corresponding author on reasonable request.

## Abbreviations

ARF: Acute renal failure
ALT: Alanine aminotransaminase
AST: Aspartate transaminase
BUN: Blood urea nitrogen
CK: Creatinine kinase
LC: Liver cirrhosis
LDH: Lactate dehydrogenase
